# Manoeuvring Across Academia in National Socialist Germany: The Life and Work of Devendra Nath Bannerjea

**DOI:** 10.1007/s00048-023-00363-0

**Published:** 2023-08-02

**Authors:** Maria Framke

**Affiliations:** Universität Erfurt, Erfurt, Germany

**Keywords:** India, National Socialism, Germany, Knowledge production, Precarity, Indien, Deutschland, Nationalsozialismus, Wissensproduktion, Prekarität

## Abstract

The article investigates the possibilities and limits for the academic Devendra Nath Bannerjea to find employment in National Socialist Germany by producing—what he imagined to be—useful knowledge for the state. Bannerjea, who came from the Punjab in northwestern India via London, Geneva and Rome to Berlin, defies neat categorization. He was neither a National Socialist scholar, nor can he be solely understood as an Indian anticolonial nationalist. In the more than four decades he spent in Europe, Bannerjea appeared in many different roles—as an anticolonial rebel, false diplomat, researcher, and endeavouring professor. Despite his employment in different educational institutions, his publications, and his political and academic networks, he remained a second row intellectual and political activist. His activities led to repeated conflicts, first with British and later Nazi authorities, because of his radical ideas and claims to intellectual egalitarianism on the one hand, and, even more often, because of his ‘creative’ efforts to improve his precarious living conditions on the other.

The article explores the relationship between knowledge production and National Socialist state politics through the lens of Bannerjea’s life, focussing on the exchange of resources between Bannerjea and the National Socialist apparatus. Against the backdrop of the social circumstances of his livelihood, it investigates the knowledge produced by Bannerjea and the rewards he received from the National Socialist regime in return.

## Abstract (de)

Der Artikel untersucht die vielfältigen Bemühungen des Akademikers Devendra Nath Bannerjea im nationalsozialistischen Deutschland, durch die Bereitstellung von nützlichem Wissen für den Staat, Arbeit zu finden. Bannerjea, der aus dem Punjab im Nordwesten Indiens stammte, war über London, Genf und Rom nach Berlin gekommen. Er lässt sich nur schwer (politisch) verorten: weder war er ein nationalsozialistischer Wissenschaftler, noch kann er vorrangig als indischer antikolonialer Nationalist verstanden werden. In den mehr als vier Jahrzehnten, die er in Europa verbrachte, nahm er ganz verschiedene Rollen, als antikolonialer Rebell, falscher Diplomat, Wissenschaftler oder Professor, ein. Trotz seiner Anstellungen in verschiedenen Bildungsinstitutionen, seiner Publikationen und politischen und wissenschaftlichen Netzwerke blieb Bannerjea ein akademischer Außenseiter und politischer Aktivist der zweiten Reihe. Seine radikalen Ideen und sein Anspruch auf intellektuellen Egalitarismus, und mehr noch seine ‚kreativen‘ Bemühungen die eigenen prekären Lebensumstände zu verbessern, führten zu wiederholten Konflikten, zunächst mit britischen und später mit deutschen Behörden.

Bannerjea’s Leben und Arbeit werden hier herangezogen, um die Beziehung zwischen Wissensproduktion und Politik des nationalsozialistischen Staates zu untersuchen. Dabei fokussiert der Beitrag auf den Austausch von Ressourcen zwischen Bannerjea und den nationalsozialistischen Behörden und fragt unter Einbezug seiner Lebensumstände, welches konkrete Wissen Bannerjea lieferte und was er für seine Expertise vom Regime erhielt.

Against the backdrop of the changing global conditions of the Second World War, Franz Alfred Six, head of Department VII of the *Reichssicherheitshauptamt* (Reich Security Main Office) and of the *Deutsches Auslandswissenschaftliches Institut* (German Institute for the Study of Foreign Countries) argued for a more pronounced academic engagement with colonial India in early 1942. Predicting the breakup of the British Empire and the emergence of an independent India, Six felt that Germany needed specialized and detailed knowledge about the subcontinent to ensure fruitful future political, economic and intellectual relations with the country. He thus urged the *Reichsministerium für Wissenschaft, Erziehung und Volksbildung* (Reich Ministry of Science, Education and National Culture) to establish a new chair for regional studies of India in Berlin. According to Six and others, the right man for the job was Ludwig Alsdorf.^1^ Alsdorf’s reputation as an expert on modern India, however, did not go unchallenged—his rival, the Indian intellectual Devendra Nath Bannerjea who had been hired to lecture on India at the *Auslandswissenschaftliche Fakultät* (Faculty for the Study of Foreign Countries) in Berlin in 1940, vigorously demanded the newly established chair for himself.

What could be easily dismissed as an ordinary professional squabble between two rival scholars provides us with a fascinating case study of the possibilities and limits for academic outsiders to work in National Socialist Germany by producing—what they imagined to be—useful knowledge for the state. Bannerjea, who led a “transboundary life” that brought him from the Punjab in northwestern India via London, Geneva and Rome to Berlin, defies neat categorization (Herren 2013). He was neither a National Socialist scholar, nor can he be solely understood as an Indian anticolonial nationalist. In the more than four decades he spent in Europe, Bannerjea would emerge as many avatars—anticolonial rebel, false diplomat, researcher, and endeavouring professor. Despite his employment in different educational institutions, his publications, and his political and academic networks, he remained a second row intellectual and political activist. His activities led to repeated conflicts, first with British and later Nazi authorities, because of his radical ideas and claims to intellectual egalitarianism on the one hand,^2^ and, even more often, because of his ‘creative’ efforts to improve his precarious living conditions on the other. To secure his economic stability and, ideally, a position of status, Bannerjea embarked upon long and often unnerving application processes for both open positions and posts still to be created. In his applications, he not only invented qualifications and gave incorrect references, but also—with a strong sense of entitlement—claimed more than once that a position had supposedly been promised to him.

On the one hand, research on German-Indian entanglements during the 1930s and the Second World War has demonstrated the heightened engagement of Indian circles with National Socialism. While Indian politicians, intellectuals and media studied and discussed certain aspects of the new German regime’s economy and society as reference models for a future Indian nation-state, they more often exhibited criticism regarding its foreign and racial policy (Framke 2013; Flora n.d.; Louro 2020; D’Souza 2000; Zachariah 2015). On the other hand, recent research has foregrounded the close interplay of cultural initiatives, knowledge production and political aspirations that informed German-Indian entanglements (Manjapra 2014; Liebau 2020; Oesterheld 2010; Sabastian 2018; Roy 2021). The following discussion adds to this literature by exploring the relationship between knowledge production on India and National Socialist state politics from the perspective, and through the work, of an ‘academic outsider’—a position that not least resulted from Bannerjea’s contested race and nationality in Nazi Germany.

Despite imperial reverberations, Weimar Germany was generally a welcoming space for non-Western students, scholars and anticolonial nationalists (Kuck 2014). This space, however, tightened after the National Socialists came to power. Their racial theories demeaned and excluded everyone who did not fit their category of ‘Aryan’, fuelling an atmosphere of open racial hatred and a soon-deadly antisemitism. Although these developments aggravated the everyday lives of Indians in Germany, the newly implemented discriminatory racial laws neither barred Indians from studying nor finding employment at German academic institutions (see also below). Yet, in response to the changed circumstances, the number of Indians residing in Germany dropped drastically. Germany’s foreign policy considerations ensured that the conditions of those who continued to live, work and study under Nazi rule remained relatively stable during the second half of the 1930s and the Second World War. With Britain as its enemy, Berlin now supported Indian anticolonial aspirations in hopes of stirring up trouble in the British Empire. In line with this strategy and despite India’s entry into the war on the side of the Allied powers, Indians living in Germany were not interned as enemy aliens during the war (Weidemann 1996: 240–241; Brückenhaus 2017: 189).

This was the world in which Bannerjea moved—the world that defined the possibilities and limits of his work. To explore the relationship between knowledge production and National Socialist state politics through the lens of Bannerjea’s life, the discussion here builds on Mitchell Ash’s work, which has demonstrated how politics and science each served as a resource for the other in twentieth-century Germany. Starting with the observation that the resources exchanged could be of “cognitive-conceptual, institutional, financial or rhetorical nature” (Ash 2010: 16), this article analyses the exchange of resources between Bannerjea and the National Socialist apparatus. To this end, it explores his concrete contributions—the production of knowledge—for the National Socialists and asks what he in turn gained for his expertise from the regime. The article provides a short sketch of Bannerjea’s life before 1933 to carve out some more lasting trends in his work biography. Following this, the article analyses Bannerjea’s stay in Nazi Germany with a focus on three different forms of knowledge production: his teaching, his writings, and the knowledge/expertise on India and Indians he offered directly to different National Socialist state institutions.

## The Complex Life of Devendra Nath Bannerjea

Devendra Nath Bannerjea led a complex, precarious, and intriguing life. Although his curriculum vitae contains various uncertainties and gaps, we can say that Bannerjea was born in the province of Punjab, in the northwestern part of British India in the late 1880s. The available sources provide hardly any insight into his family background apart from the fact that his father was at one time employed in the Education Service of Punjab.^3^ After receiving his graduate and postgraduate education at the Ferman Christian College and the Government College, both in Lahore, he worked for two or three years as a teacher of political economics and English literature at a community college in Lahore. Before the outbreak of the First World War, Bannerjea came to London and was admitted for a BLitt degree at Christ Church College, Oxford University. Whether he finished his studies at Oxford with a degree is unclear. We know for sure that in 1934 he received a Ph.D. from the University of Leipzig.^4^ In 1914, Bannerjea signed up for Gandhi’s Indian Field Ambulance Training Corps in London. The voluntary humanitarian initiative provided medical assistance to wounded and sick Indian soldiers fighting in Europe on the side of the Allied powers (Hunt 1993: 162–171; Devji 2018: 192–198). Bannerjea worked as an interpreter at the model hospital complex for British Indian troops in Brighton (Hyson & Lester 2012). His service, however, ended prematurely after being “suspected of attempting to tamper with the loyalty of the Sepoy patients”.^5^

In the following years, Bannerjea was forced to work at different jobs to eke out a living. Among other positions, he assisted the radical Indian nationalist Bal Gangadhar Tilak who had come to Great Britain in 1918 to represent India at the peace conference at Versailles.^6^ Around 1917, he began writing to his future wife Hilda M. Howsin, an ardent supporter of Indian independence who had published a book, *The Significance of Indian Nationalism* in 1909 (Howsin 1909), and had been interned on the basis of ‘hostile associations’ under the Defence of the Realm (Consolidation) Act at Aylesbury prison since September 1915. Her internment resulted from the British discovery of her contact to the ‘notorious’ revolutionary Virendranath Chattopadhyaya and the government’s suspicion of her involvement in a Hindu-German conspiracy case (Popplewell 1995: 224–225; Barooah 2004: 68 and 136). After her release from prison in August 1919, Bannerjea and Howsin began working together. In 1920 and 1921, both contributed several articles to the magazine *The Venturer*, published by the British Fellowship of Reconciliation. While the themes Bannerjea and Howsin engaged with were diverse, reaching from larger international and imperial politics and history to matters of education, law, administration and the independence movement in colonial South Asia, their contributions fundamentally and consistently questioned the foundations of (British) imperialism and the post-war international order (Howsin 1920a, b; Bannerjea 1920, 1921a, b). Thus, when Earl Reading was appointed the new Viceroy of India in 1921, Bannerjea composed an open letter to him, arguing keenly for a change of British policy towards India. In the letter, published in the *Venturer*, Bannerjea described India’s national aspirations to self-determination and democracy and proposed an imperial conference in Delhi to facilitate discussions between the colonial government and Indian nationalist politicians about India’s future (Bannerjea 1921c: 237–238). The situation in his homeland remained a topic Bannerjea wrote about and engaged with in the years to come.

In 1922, Bannerjea and Hilda, now married, left Britain and moved, after a short stay in Baden-Baden, Germany, to Berlin. During this time, Bannerjea also became a member of the Committee on Intellectual Co-Operation of the League of Nations in Geneva (for the Committee see, Laqua 2011).^7^ The particularities of his appointment remain nebulous. The League was convinced it had appointed a professor of political economy from the University of Calcutta named Bannerjea. After the League realised in September 1923 that it had appointed the wrong Mr. Bannerjea, the whole episode became a matter of huge annoyance and embarrassment to the British government, especially the India Office. The latter tried to remove him from the Committee and replace him with a more suitable—meaning politically less dubious—candidate; though this proved difficult (Schmidt 1994: 231–235). Bannerjea, while rejecting the British government’s right to get involved, tenaciously and repeatedly argued his case with British officials and League of Nations deputies, stressing that his appointment was based upon his intellectual merits. When one reads through the files of this minor scandal, two facets feature prominently: first, it seems that Bannerjea took great pains to stick as closely as possible to the truth, or his version of the truth. Second, Bannerjea made several efforts to solicit support from sympathetic colleagues, as well as from prominent individuals, such as Prime Minister Ramsey MacDonald and Lord Robert Cecil, the president of the British League of Nations Union—pointing to his abilities as a networker.^8^ Over the next two decades, Bannerjea would hark back to both strategies in subsequent crises. In addition, the episode portends Bannerjea’s firm belief in the importance of education and his efforts to make non-Western intellectual contributions better-known in Western circles. Bannerjea participated in the committee’s work, providing valuable suggestions regarding the development of an international university, the financial and legal support of international (non-Western) students, and the wider use of Esperanto as a universal language. He also used his position to publicly argue for mass education in India and an extended programme of university exchanges for professors (Schmidt 1994: 238–239; Bannerjea 1923).^9^

After his final dismissal from the Committee on Intellectual Co-Operation in March 1924, Bannerjea surfaces again in the archives in 1927. By this time, he had settled in Italy and partly made his living by working on a temporary basis for the US-delegate Asher Hobson at the International Institute of Agriculture in Rome.^10^ It seems that he faced rather precarious circumstances, as he tried vigorously to convert his temporary contract into a permanent position at the institute. Although his application was supported by various factions and influential people, such as labour politician Charles Trevelyan, it failed due to the opposition of the India Office, which had to agree to the appointment. The India Office refused, pointing not only to the previous incident at the League of Nations, but also to his wife’s past internment.^11^ His inability to secure a permanent position and thus a stable income seems to have resulted in yet another relocation: Bannerjea returned to Germany. At the recommendation of the German Foreign Office, Bannerjea received a teaching assignment at the *Seminar für Orientalische Sprachen* (Seminar for Oriental Languages) at the University of Berlin in 1928 or 1929.^12^ Over the next one and a half decades—during the National Socialist period and the Second World War—Bannerjea remained employed in various departments of the university. In April 1945, he left Berlin with his wife and reached Bamberg University via Bohemia, where he began to teach English literature in spring 1946, an appointment he held until 1950.^13^ Bannerjea died on 10 December 1954 in Bamberg. After his death, Hilda returned permanently to Britain.^14^

## Moving in and out of the Precariat: Bannerjea’s Life as an Academic Outsider

Michael Goebel has recently suggested that scholars engage more seriously with the social history of anticolonial activists, with their mundane concerns and everyday life, in regard to employment, (romantic) relationships and encounters with racial prejudices (Goebel 2016). This interjection seems all the more important for transboundary actors like Bannerjea, whose financial circumstances consequently determined his “scope of possibility”. This framework, used by Svenja von Jan to analyse the impact of socio-economic factors on the life trajectories of subaltern South Asian migrants to Europe, can equally be applied to the life and work histories of elite South Asians in European societies (von Jan 2019). Their ability to tap into certain resources and to create networks, as well as their efforts and willingness to ‘perform’ within, accept, or reject the political system of their (chosen) country of residence did affect their socio-economic situation and the scope of possibility in their lives and vice versa. This observation might seem self-evident; however, most scholarly works on the lives of South Asian intellectuals, elite revolutionaries and nationalists in Europe have thus far only given the issue a cursory glance. Although they mention the precarious financial circumstances of the actors in question, these works neither expand on the impact of those economic and social factors on the room for manoeuvre, nor on the question of how exactly the relationship between everyday concerns and intellectual production worked (for one example that does engage with these questions, see Laursen 2023).

Devendra Nath Bannerjea taught at the University of Berlin from 1928/29 to 1945 on a precarious salary of 85 to 200 Reichsmark per month during the 1930s.^15^ Bannerjea—who during this period was also supporting his wife financially—repeatedly asked for a raise and additional allowances, which would enable him to deal with illnesses, pay rent or move his belongings from Rome to Berlin. During these years, creditors attempted to seize his salary twice, albeit without much success as his income was legally too low to be confiscated.^16^ While his requests for a higher salary repeatedly failed in the years before 1940, Bannerjea managed to receive several non-recurring grants from the university.^17^

In Bannerjea’s case, his precarious situation motivated his efforts to secure a permanent position. In the first six years of his stay in Germany, he taught *Indische Realien* (a course dealing with practical subjects relating to India) at the *Seminar für Orientalische Sprachen* and courses on Indian language and culture at the *Institut für Auslandsstudien* (Institute for Foreign Studies). After the director of the *Seminar für Orientalische Sprachen*, Hans Heinrich Schaeder, suggested that the university forego any further employment of Bannerjea as other colleagues were able to cover the needed India expertise, his assignment was not renewed in 1935.^18^ In his letter to the Reich Ministry of Science, Education and National Culture, Schaeder—without giving details—pointed to existing concerns regarding Bannerjea’s personality and argued that the money saved could be spent on other necessities. Schaeder’s recommendation put Bannerjea in a tight spot and triggered a prolonged correspondence in which various people from his academic, cultural, and political circle intervened on Bannerjea’s behalf. Amongst his advocates were Professor Ernst Schultze, Bannerjea’s Ph.D. supervisor and director of the *Weltwirtschaftsinstitut* at the *Handelshochschule* (Institute for World Economics at the Commercial College) in Leipzig, Dr. Walter Lierau, a retired lieutenant colonel working for the Foreign Office and SS officer, Dr. Oskar Ritter von Niedermayer, a German officer and associate professor at the University of Berlin for *Wehrgeografie und Wehrpolitik* (Defence Geography and Policy) and the author and translator Franz Fromme, who was a staunch proponent of National Socialism.^19^ They not only supported Bannerjea’s case by pointing to his academic credentials, but also by describing him as reliable and politically unobjectionable. Dr. Lierau, who had known Bannerjea for twelve years, for instance, emphasised, “from the outset in Italy, he showed a great understanding for Fascism and later, in Germany, for the Hitler movement. He was particularly interested in this movement in view of its philosophical principles and regarding the agrarian situation in India.” Lierau was convinced that Bannerjea would work at the University of Berlin in compliance with National Socialist principles.^20^ Bannerjea’s support network lifted him into a new paid teaching position at the *Staatswissenschaftliches Seminar* (Seminar for Public Policy) for *Übungen zu indischen Realien* (tutorials on *Indische Realien*) in late July 1935.^21^ At the same time, he continued teaching at the *Ausland-Hochschule* (Foreign Studies College). As the assignment at the university did not provide any permanency, Bannerjea continued to attempt to stabilize his position.^22^

He received another chance to do so in 1940, when the *Ausland-Hochschule*, the former *Seminar für Orientalische Sprachen*, merged with the *Deutsche Hochschule für Politik* (German College for Politics) to form the *Auslandswissenschaftliche Fakultät* (Botsch 2017, 2006). This move was facilitated by the Reich Ministry for Science, Education and National Culture which championed the *Auslandswissenschaften* (knowledge on foreign regions). At the suggestion of Franz Alfred Six, Bannerjea began teaching at the new faculty in 1940 in the department of *Landes- und Volkskunde Großbritanniens und des Weltreiches* (Regional Studies of Great Britain and the Empire), where he lectured on India. Six argued that Bannerjea was very well-suited for the job owing to his extensive teaching record and his reputation at the *Ausland-Hochschule*. As a high-standing SS functionary and Himmler’s stalwart, Six’s support for Bannerjea could indicate that the latter enjoyed positive relations with the SS by this point. In addition, Bannerjea enjoyed the trust of the Foreign Office, and thus Six not only recommend him for employment, but also advocated for him to receive a salary increase.^23^ The assignment that finally provided Bannerjea with a steadily increasing income lasted until the end of the winter semester 1942/43 when it was revoked, and Ludwig Alsdorf took over the teaching of modern Indian studies at the faculty. One year later, the *Auslandswissenschaftliche Fakultät* created a new chair for *Volks- und Landeskunde Indiens* (Regional Studies of India) to which Alsdorf was appointed.^24^ This appointment did not go unchallenged by Bannerjea, who had repeatedly lobbied the university, as well as various government offices, such as the Reich Ministry for Science, Education and National Culture, the Reich Ministry of Foreign Affairs and the Foreign Office to grant him the chair.^25^ However, his direct and at times unnerving demands remained unsuccessful, as his behaviour alienated him from university and government authorities.^26^ In his efforts to secure the professorship, Bannerjea, in the words of one Nazi official, “did not show the necessary restraint that in the current circumstances should be imposed on foreigners in Germany”.^27^ Six, who had previously supported him, now felt that Bannerjea lacked the necessary distance in regard to contemporary developments in India.^28^ Despite these critiques, Bannerjea received a new teaching assignment, this time for languages and *Indische Realien* at the *Indologisches Seminar* (Indological Seminar) from the summer semester 1943 onwards. With a salary of 4,200 Reichsmark per year, this position paid much better than his previous appointments and finally put him on a more secure financial footing.^29^

Investigating Bannerjea’s employment trajectory in Germany reveals that his room for manoeuvre was rather tight. His economic circumstances repeatedly compelled him to request a position from the University of Berlin. After 15 years of precarity in Germany, he finally managed to secure a certain measure of financial stability through his last teaching assignment. Yet, his employment history simultaneously reveals that Bannerjea ably managed to expand his room for manoeuvre by establishing and deploying support networks and—as the next section will show—by offering his knowledge on India and Indians to German state authorities.

## Producing Knowledge in and for National Socialist Germany

Bannerjea’s employment at the University of Berlin during the entire Nazi period indicates that German government and academic authorities deemed his work to be useful. Bannerjea’s expertise on South Asia is reflected in the subjects he taught at the university. Course titles from 1933 to 1945 highlight the wide thematic span he covered in his classes, comprising—in addition to Hindustani—the politics, culture, economy, education, and history of India.^30^ Several of his lectures and tutorials dealt with contemporary issues, such as the Indian independence struggle, the Indian National Congress, Mahatma Gandhi, the 1935 Government of India Act and India’s relationship to Britain.^31^ The course titles also reveal that Bannerjea adjusted his teaching according to the audience. Thus, during his time at the *Auslandswissenschaftliche Fakultät*, he for instance lectured on German-Indian cultural and economic relations and on the war and its meaning for India’s political future.^32^ Such courses were connected to the larger aim of the *Auslandswissenschaftliche Fakultät* to not only instruct the next academic generation, but also to train and educate (future) employees of the German secret services and to create valuable content for ideologically appropriate entertainment via media, broadcasting and censorship (Roeske 1992: 1–3; Botsch 2017). Whether India studies in Berlin succeeded in contributing to this aspiration is debatable, as for instance only a very small number of students enrolled in the Indian language classes at the *Auslandswissenschaftliche Fakultät* (Framke 2014: 112–114). Nonetheless, it is safe to say that Bannerjea provided knowledge that was wanted.

While Bannerjea taught continuously, he published less frequently from the mid-1930s onwards. Beginning in the late 1910s, he had written both academic and journalistic articles, essays, reviews, and two monographs—*India’s Nation Builders* (1919) and *Das indische Bauerntum unter britischer Herrschaft* (The Indian peasantry under British rule, 1934a). His writings focused on four broad themes: contemporary world politics with a particular interest in international and imperial affairs, educational politics, agriculture, and—most importantly—India’s political status (Bannerjea 1919, 1921a, b, 1923, 1926a, b, 1927). Promoting India’s case for freedom in his publications, Bannerjea pointed repeatedly and unequivocally to the various wrongs of British rule in India, such as the impoverishment of the colonized country due to the financial and administrative system, the frequent famines, the neglect of the health, social welfare and educational sector, and the policy of divide and rule (Bannerjea 1928: 60–64; Meston & Bannerjea 1928: 1–16). His criticism of British rule culminated in an article, published in *Current History* in 1933, in which he emphasised that Indian self-government could not be achieved through any cooperation with the present government (Bannerjea 1933: 169–175). His efforts to make India’s case more popularly known were directed towards British, American and—beginning in the late 1920s—German audiences. Yet, after 1933, it seems that he stopped publishing on a regular basis (Bannerjea 1934a, b, 1938), though it is possible that some of his later publications have been lost. This cessation of critiques of British rule in India might have been influenced by the Nazi regime’s pro-British stance during the 1930s, which discouraged open anti-British propaganda in Germany (Brückenhaus 2017: 180).

Moreover, the decline in published output did not mean that Bannerjea had stopped writing. In 1934, he drafted a journalistic piece for Indian audiences that foregrounded a culturalist argumentation of India and Germany’s affinity with each other and emphasised, more so than in the past, their racial ties. In the article, published in two nationalist Indian dailies, *The Bombay Chronicle* and the *Amrita Bazar Patrika*, Bannerjea claimed: “Germans of all classes and walks of life welcome us as Aryan brothers, they identify themselves with what is permanent and creative in our life and culture; they sympathise with our struggle for complete self-expression” (Bannerjea 1934b: 18; Bannerjea 1934c: 5). Bannerjea went on to dismiss the reporting in English, Swiss and Austrian newspapers on efforts by the National Socialist government to pass discriminatory legislations against Indians—for instance, the prohibition on marriages or social relations with Germans. The suggestion that the German government understood Indians not as Aryans or as racially inferior seemed absurd to him. To counter these reports, he explicitly pointed to a Reuters interview with Wilhelm Frick, the German Minister for the Interior, in which he had allegedly stated that the “German people wish to maintain themselves racially pure, but they certainly do not look upon any Asiatic people as an inferior nation, and they are fully aware that some of them have an ancient and progressive culture” (ibid.). Although Bannerjea rightly emphasised that racial laws interdicting marital and sexual relations between Germans and ‘non’-Germans had not been enacted, his account neglected to mention that such ideas had been proposed by the Prussian Justice Minister Hanns Kerrl and his Undersecretary Roland Freisler. Bannerjea’s take on an Indo-German Aryan brotherhood and German racial imaginations clearly ignored the opinion of leading National Socialists who viewed Asians as racially inferior (Gruchmann 1983: 419; Framke 2013: 118–120). In *Mein Kampf*, Adolf Hitler vilified Indian nationalists as “Asiatic imposters”. In line with an understanding of a common racial affinity between German and British people, he had also endorsed British colonial rule over India (Hitler 1932: 747). These and other repeated derogatory remarks sparked prolonged discussions and feelings of indignation in India. Indian criticism, however, often did not question the very idea of a race hierarchy but repudiated the inferior position ascribed to Indians in National Socialist thinking (Framke 2013: ch. 5.1). Mirroring Bannerjea’s views, these voices thereby attributed a high racial status as Aryans to Indians. In its praise for Germany, Bannerjea’s article in *The Bombay Chronicle* and the *Amrita Bazar Patrika* likewise overlooked the daily racist experiences of non-white people in Hitler’s Germany, a problem also faced by Indian residents and students. Their experiences of racist discrimination, in addition to the maltreatment and expulsions of a group of Indians from Germany in response to the anti-left-wing agenda of the Nazis, had been critically reported in Indian newspapers since 1933. The situation as a whole also caused Indian student organizations in Germany as well as nationalist politician Subhas Chandra Bose to lodge their protest with the German government in spring 1934 (Voigt 1971: 40–41; Kuhlmann 2003: 51–52; Brückenhaus 2017: 169–178). The critical reporting and the Indian protests did not go completely unheard and spurred the German Foreign Office and the *Deutsche Akademie* (German Academy) to reassure Indians of the friendly and safe conditions for them in Germany. In doing so, both institutions strove to keep the cultural, political—and most importantly—economic relations between Germany and India stable (Voigt 1971: 40–41; Kuhlmann 2003: 51–52; Thierfelder 1933).^33^ Against this backdrop, Bannerjea’s silence on the issue is conspicuous, even more so since he made a strong case for strengthening the Indo-German friendship, not only in commercial but first and foremost in educational terms. Pointing to available scholarships for Indian students to continue or complete their studies in Germany, Bannerjea urged his compatriots to further increase the number of Indian students coming to Germany and to participate in a systematic academic exchange between the two countries (Bannerjea 1934b: 18).^34^ Bannerjea’s decision to voice his opinion at a time when German-Indian relations were disrupted by the virulent racism of National Socialism can be understood as a conscious effort to help stabilizing these very same relations. Nonetheless, whether his interjection in the debate surrounding Indian-German relations had been commissioned by the German Foreign Office remains uncertain.

Over the course of the following decade, Bannerjea also worked on two larger academic projects. In addition to his habilitation (a second qualification thesis required in Germany to become a professor) on *Die Volkswirtschaftlichen und Staatsrechtlichen Grundlagen des Moghulen Kaiserreichs in Indien, 1526–1768* (the economic and constitutional principles of the Mughal Empire in India, 1526–1768), he also prepared a biography on Mohandas K. Gandhi. For different reasons, however, neither work was ever published. In 1938, Bannerjea was admitted to complete his habilitation at the University of Berlin. It took him until January 1942 to submit the work, which he did at the University of Munich. Bannerjea explained his change of academic institution during the habilitation process with a lack of support in Berlin, where he failed to find an academic supervisor. From a letter from Six to the Reich Ministry of Science, Education and National Culture, we know that the habilitation was first submitted to the Faculty of Law and Public Policy at the University of Berlin, which did not accept the work as its content did not reflect the faculty’s disciplinary focus. A second submission to the Philosophical Faculty of the University of Berlin was also declined; the letter provides no explanation for this rejection.^35^ Whether or not the habilitation was accepted in Munich is also unclear, but given that Bannerjea stopped mentioning, it is likely that he was not successful in Munich either.^36^

The Gandhi biography, completed by Bannerjea in 1942, did not serve to further his academic qualifications, but was a commissioned work. Franz Josef Furtwängler, who worked in the *Sonderreferat Indien* (Special Office India) at the Foreign Office, served as editor of the book, which was expected to be published by the Vowinckel Press. The *Sonderreferat Indien,* a special subsection of the Foreign Office, was set up in spring 1941 with the arrival of the Indian nationalist Subhas Chandra Bose (Kuhlmann 2003: 158–165). The book contract with Vowinckel did not mention any involvement of the Foreign Office, however, in the following months Bannerjea repeatedly pointed to the government office as the commissioning party—a claim that its employees emphatically denied.^37^ After the manuscript’s submission, it was sent to a reviewer—Ludwig Alsdorf—who did not approve of the work and judged it completely unusable. The unfolding controversy around the manuscript bristled with personal defamations, questions regarding its whereabouts after being reviewed, what to do with it and whether Bannerjea should receive the contractually agreed upon payment. The conflict between the Foreign Office, the publishing house, Bannerjea and, eventually, Bannerjea’s lawyer lasted until 1944. In the end, Bannerjea was paid in full for his work, but his manuscript was never published.^38^

Bannerjea’s cooperation with National Socialist institutions, visible in his efforts to get the Gandhi biography published, echoed his earlier efforts to tap in a network of supporters in order to secure teaching assignments. Between 1933 and 1945, Bannerjea repeatedly offered his expertise on India and on the Indian diaspora community in Berlin to the regime. Pre-dating the Hitler government, several initiatives involved his efforts to foster a political, economic, and cultural rapprochement between Germany and India. In 1930, for instance, he founded a German-Indian Society.^39^ One year later, he stressed the intellectual, cultural, and racial affinity of the two countries in a talk and argued for closer Indo-German cooperation. He concluded his remarks, published in the journal *Hochschule and Ausland*, by saying:

“We see that understanding and mutual interest have racial and historical causes: They are based on the fact that both countries have in the past made valuable contributions to the good of culture and that they are destined to serve the progress of humankind in the future. And as German scholars have found the root of ancient India’s life and have delved into its literature, so we hope that the next generation in Germany will dedicate itself to the life and problems of the new India with the same affection and depth.” (Bannerjea 1931: 9)^40^

Bannerjea’s endeavours to strengthen Indian-German linkages connected with, but later also rivalled similar efforts by other South Asian anticolonial actors, intellectuals and politicians living in or traversing interwar Europe (Manjapra 2014; Sabastian 2018; Framke 2016: 61–65; Kuhlmann 2003). In July 1931, when the well-known nationalist and former President of India’s Central Legislative Assembly Vithalbhai J. Patel visited Berlin, Bannerjea was one of his hosts. As president of the Indian National Union of Berlin—one of the various, often transient, initiatives that sprang up in Central Europe in the 1920s and 1930s—Bannerjea introduced Patel to the Indian community and a wider German audience during a tea party in the Eden Hotel and at a dinner two days later in the Hindustan House. On both occasions and during a lecture organized at the English Seminar of the University of Berlin, Patel spoke about the Indian anticolonial struggle and stressed the Indian National Congress’ leading role. Although he did not publicly call for political support from Germany, Patel underscored the significance of Indo-German economic relations (From our correspondent 1931a: 7, 1931b: 9; From a correspondent 1931: 1). Passing through Germany again a few months later, Patel contacted the German Foreign Office with Bannerjea’s help.^41^ Patel’s visits in Berlin may have motivated Bannerjea to plan what would have been a brilliant coup for Indo-German relations, namely a visit by Mohandas K. Gandhi to Berlin and other German cities following Gandhi’s attendance at the 1931 Round Table Conference. In support of this plan, Bannerjea received the support of prominent German Indophiles, such as Count Herman Keyserling and Franz Joseph Furtwängler; however, Gandhi’s visit did not materialise in the end.^42^

With the National Socialist seizure of power in 1933, Bannerjea’s work for Indo-German rapprochement continued; his German-Indian Society, for instance, remained operational until 1937. His relationship with the larger Indian anticolonial movement, however, soon became estranged. With Subhas Chandra Bose’s visit to Germany in summer 1933, a new actor emerged on the scene, who, due to his prominence, readily gained access to the German Foreign Office. After an unsuccessful attempt to reconcile different factions in the Indian community in Berlin, Bose established a new organization, called the Indian Students’ Association. Bose seems to have initially enlisted Bannerjea’s support for his activities, although there are indications that the latter understood Bose’s intervention as competition to his own attempts to strengthen Indo-German links. Within a few months, Bannerjea’s relations with the Indian Students’ Association turned sour over an incident that involved him providing the German authorities with information about the (alleged) communist leanings of three members of the Indian community in Berlin. Once Bannerjea’s statement became public, the Indian Students’ Association declared him an outcast in February 1934.^43^ As we do not know of any cooperation with Indian nationalists in Germany in the following years, it seems that this incident isolated Bannerjea within the community (Weidemann 1996: 250).

Yet, this had not been the first time that Bannerjea collaborated with German authorities. After their accession to power, the National Socialists implemented an anti-left-wing agenda that, as Daniel Brückenhaus has shown, targeted non-German anticolonialists living in Germany. One victim of this policy was the Berlin-based Indian student Monindra Kumar Sen who was expelled from the country after the police accused him of being politically unreliable (Brückenhaus 2017: 169–177).^44^ The police assessment was based on several denunciatory reports—one of which came from Bannerjea, another from his wife Hilda.^45^ These reports, however, did not become public, as would be the case with the three ostensible communists. Bannerjea’s rationale for both denunciations is unclear. Whether he was a convinced National Socialist, as one could conclude from several antisemitic remarks and the admiring language for Hitler in his letters, or his cooperation with the Nazis was instead driven by his longstanding efforts to carve out a living and a position of status, remains unsolved. What emerges from the files is that Bannerjea continued to report on other Indians with (alleged) communist leanings and Indians who had spoken out critically against the Hitler regime.^46^ These activities made him politically reliable and worthy of support by members of the government and the university establishment in his efforts to secure a permanent position. At the same time, the Gestapo requested that he stop his denunciations, as they felt his reports were based more on personal differences than on provable offences according to the National Socialist ideology and law.^47^

These episodes demonstrate that Bannerjea’s connections with the Nazi regime went beyond the realm of academic knowledge production in the form of teaching and writing. Furthermore, these were not the only occasions at which he offered his expertise on India and the Indian diaspora community to the National Socialist state. Beginning in spring 1939, Germany had begun to produce special radio programmes broadcasting first anti-British and then also anti-French messages for Arabic-speaking listeners and colonial audiences. By pointing to the hypocrisy of Western democracies maintaining their colonial possessions and mandates and thereby demonstrating Nazi sympathies with nationalist aspirations, these programmes were deployed to incite unrest in these areas and thus weaken the war effort of Germany’s enemies (Brückenhaus 2017: 191–192; Herf 2011; Goldbaum 2016). In the broadcasts produced for Indian listeners, British rule in India was compared with German politics in order to present National Socialism as a model for the subcontinent (Kuhlmann 2003: 76). Radio propaganda thus emerged as an important aspect of Germany’s foreign policy during the war. Bannerjea became part of this propaganda effort in 1940, when he joined the *Rundfunkpolitische Abteilung* (the broadcasting section) of the Foreign Office. For three months, he reviewed German broadcasts in Hindustani, before he was assigned to the *Seehaus* unit, the news monitoring section of the Foreign Office. During this second assignment, which continued until June 1941, Bannerjea listened to intercepted allied Hindustani broadcasts from Delhi and London. Owing to his teaching obligations, as well as ongoing health issues, Bannerjea fulfilled his tasks for both sections only irregularly. In consequence, state authorities released him from further duties.^48^

Despite his longstanding relationship with the German Foreign Office, dating back to the 1920s, Bannerjea did not participate in any significant propaganda programmes during the war. After his arrival in Germany in April 1941, Subhas Chandra Bose established the *Zentrale Freies Indien* (Free India Centre) with the assistance of the German Foreign Ministry. The centre’s anticolonial programme vigorously criticized British imperialism, yet its radio programmes and publications also contained pro-Axis propaganda. For the work, Bose recruited Indians still living in Germany as well as compatriots from other parts of Europe who had fled the country during Nazi rule (Roy 2022; Kuhlmann 2003: 158–165 and 177–179; Brückenhaus 2017: ch. 6). Bannerjea was not amongst the recruits. The sources do not tell us whether his exclusion derived from his controversial position in the Indian diaspora community or from his workload at the university. All we know is that Bannerjea did not join the centre and hence did not contribute to its knowledge production.

His absence from the *Zentrale Freies Indien* did not hinder Bannerjea from turning to the Foreign Office with a suggestion to establish a new Indo-German society in May 1942 in order to strengthen mutual relations between Germany and India. To make the initiative a success, a close cooperation with the German government was needed in his opinion.^49^ Simultaneously, Bannerjea also advocated for the Foreign Office to set up an India Institute which he envisaged as an institution of academic propaganda. He justified his proposal by pointing out that, after the war, profound knowledge of India would be a dire necessity for Germany and the cornerstone for any future close cooperation between the two countries. Although the rapprochement between Germany and India had been a long-term interest of Bannerjea’s, he used the proposal to bring himself into play as a suitable candidate for the professorship that would be linked to the institute. He corroborated his suitability with his twofold expertise as an Indian “who due to his descent and nationality could impart directly knowledge of his motherland and who at the same time has the necessary education and familiarity of the scientific methods that are the custom at German universities”.^50^ Bannerjea’s proposals were taken seriously by the Foreign Office and discussed internally. In fact, his plan for an India Institute was positively evaluated by his rival Ludwig Alsdorf—except that Alsdorf stressed that only Germans could be in charge of such an institute, as the institute would be first and foremost a German concern. As far as we know, the plans did not materialise, as the political circumstances did not seem to favour such an initiative during the war years.^51^ Ultimately, the state backed a competing scheme and instead supported the establishment of a new India chair at the *Auslandswissenschaftliche Fakultät* in Berlin in 1943.

Moving beyond the macro level of academic institutions, this article has analysed the relationship between knowledge production—namely knowledge about India and the Indian diaspora—and state politics during the Nazi period through a micro-level focus on the leeway that existed for intellectual cooperation for one Indian scholar in Germany. Taking a cue from Mitchell Ash’s findings, the discussion has revealed the exchange of various resources between Bannerjea and the National Socialist authorities. Although he was an academic outsider to the National Socialist system (an argument that was repeatedly used in 1942 to stop him from becoming a professor in Germany), Devendra Nath Bannerjea managed to work and earn a living as a scholar during Nazi rule. Certain institutions of the National Socialist state regarded his scholarly output as a lecturer and to a lesser extent as a writer as ‘useful’ enough during the Second World War to employ him; these employment opportunities provided Bannerjea with a status and, more importantly, an income. Yet, his financial circumstances remained precarious for a long time, and thus created a permanent dependence on state support. To be able to secure this support, Bannerjea engaged in various extra-curricular activities, such his collaboration with the Foreign Office and the police before and during the Second World War and his manifold efforts to stabilize and strengthen Indo-German relations. In doing so, he provided India-related knowledge that seems to have served the political ambitions of Nazi Germany’s cultural, economic, and foreign policy.

Bannerjea does not seem to have overly distinguished himself from other academics of Indology and Indian studies in Berlin during the Nazi period. In addition to Bannerjea’s rival Ludwig Alsdorf, Tarachand Roy and Hermann Beythan also engaged in various forms of knowledge production that transcended their core academic discipline. By cooperating with the Foreign Office and by giving public lectures, these scholars produced popular knowledge on India—and thus a resource for the German state to achieve its economic and political objectives (Roy in this special issue; Framke 2014: 103, 112).^52^ Nonetheless, Bannerjea’s career differed from Alsdorf’s insofar as he failed to publish academic works that would have increased his scholarly reputation and might have allowed him to become a full professor.

Delineating the multiple forms of knowledge that Devendra Nath Bannerjea produced and provided to German academic and government audiences also undergirds the call to take the social circumstances of his livelihood into account. The main resources Bannerjea could hark back to, the resources that shaped his ‘scope of possibility’, were his India expertise and his ability to network. As the article has demonstrated, Bannerjea made full use of his resources: To secure a means of subsistence for himself and his family, as well as a meaningful career, Bannerjea offered his knowledge and cooperated with the National Socialist regime.

## Acknowledgements

I am indebted to the editors of this Forum issue, to the two peer-reviewers, and to Joanna Simonow and Isabella Schwaderer for their comments and criticism. I also want to thank Ole Birk Laursen for our insightful discussions about Bannerjea’s life over coffee and for sharing newspaper articles on V. J. Patel with me.

## Footnotes

1 Bundesarchiv Berlin (German Federal Archives, Berlin, hereafter BArch), R/4901/14533: Letter by Six to Reich Ministry of Science, Education and National Culture, 16. January 1942 and Note: Politische und wissenschaftliche Begründung zur Einrichtung eines Lehrstuhls für Indien, no date.

2 Writing on Indian-German entanglements in the interwar period, Ravi Ahuja has recently raised the important question of how interactions and relationships between Germans and Indians could happen on the same level “in an intellectual climate that was clearly not shaped by anything resembling cosmopolitan egalitarianism” (Ahuja 2020: 324). Yet, as we will see, Bannerjea made this claim again and again.

3 The information on Bannerjea’s CV is taken from two files, if not stated otherwise: BArch, DS800, A0003 (R4901/24156): CV of D. N. Bannerjea, no date; British Library London, Asia, Africa and Pacific Collections (hereafter BL, APAC), L/E/7/1315 File 3741: Confidential note, 1923. Both files give different years and places of birth: either 1889 in Ludhiana or 1885 in Lahore. The comparison of the two files reveals a continual problem of ambiguous dates, for instance regarding Bannerjea’s migration to Britain and his university years. For the snippet on Bannerjea’s father see, BL, APAC, L/PJ/12/2, File 949/16: Note on Bannerjea, undated.

4 Email correspondence with Faye McLeod, Keeper of University Archives, Bodleian Library, Oxford, 11. November 2021; Universitätsarchiv Leipzig (University Archive Leipzig): Phil.Fak.Prom. 02066.

5 BL, APAC, L/E/7/1315 File 3741: Confidential note, 1923.

6 BL, APAC, L/E/7/1315 File 3741: Confidential note, 1923. As the British government did not allow Tilak to go from London to Paris, he remained in the British capital where he continued to propagate the idea of India’s self-determination by distributing pamphlets, published by the India Home Rule League’s Office in London in 1918 and organising a petition campaign (Manela 2007: 163–168).

7 BL, APAC, IOR/L/E/7/1315, File 3741: Confidential note, 1923.

8 See the correspondence in: BL, APAC, L/E/7/1315, File 3741.

9 BL, League of Nations. Committee on intellectual co-operation. Minutes of the first session, Geneva, August 1st–5th, 1922, pp. 31–32 and 46; BL, League of Nations. Committee on intellectual co-operation. Minutes of the second session, Geneva, July 26th to August 2nd, 1923, pp. 13–14, 31 and 69–71; League of Nations. Committee on intellectual co-operation. Minutes of the third session, Paris, December 5th to December 8th, 1923, p. 29.

10 After the Second World War, the assets and mandate of the International Institute of Agriculture transferred to the Food and Agriculture Organization (FAO) of the United Nations.

11 See the correspondence in: BL, APAC, L/E/7/1526, File 146.

12 Universitätsarchiv Humboldt-Universität zu Berlin (University Archive Humboldt University Berlin, hereafter Uniarchiv), Personal-Akten des Lehrbeauftragten am Staatswissenschaftlich-Stat. Seminar, Dr. Devendra Nath Bannerjea: Letter by Prof. Mittwoch, Seminar for Oriental Languages to Administrative Director, 07. August 1931. The *Seminar für Orientalische Sprachen* was founded in Berlin in 1887 with the aim of teaching living languages and contemporary cultures. The cost for the department were partly borne by the German Foreign Office, which was also involved in its administration. Since the First World War, plans had circulated to remodel the department into a College for Overseas Studies. These plans were implemented in 1936 and the institution was renamed the *Ausland-Hochschule* (Lötzke & Oesterheld 1973: 2–7).

13 Uniarchiv, Lehrbeauftragter Dr. Bannerjea, Bd. II. B 45: Letter by Bannerjea to Vice Chancellor, 30. Dezember 1945; Personal correspondence with Dr. Karin Amtmann (University Archive Bamberg), 29. June 2010. According to the university’s course catalogue, Bannerjea mostly gave seminars on Shakespeare, Milton and Chaucer, but also taught courses on the history and culture of Great Britain.

14 Personal correspondence with Dr. Robert Zink (City Archive Bamberg), 07. September 2010. The information on Bannerjea’s death etc. can be found in Melderegister, Bamberg file StadtAB, Rep. C9, Nr. 58a.

15 Uniarchiv, Personal-Akten des Lehrbeauftragten am Staatswissenschaftlich-Stat. Seminar, Dr. Devendra Nath Bannerjea: Copy Prussian Minister for Science, Art and Education to Vogel, 13. July 1932; Letter to the Dean, 18. January 1937; Letter by Scharschmidt to Reich Minister for Science, Education and National Culture, 16. October 1939; Uniarchiv, NS-Dozentenschaft der Friedrich-Wilhelms-Universität Berlin, Dr. Devendra Nath Bannerjea: Letter by Bieberbach to Bannerjea, 01. August1935.

16 Uniarchiv, Personal-Akten des Lehrbeauftragten am Staatswissenschaftlich-Stat. Seminar, Dr. Devendra Nath Bannerjea: Letter by Liesenberg to University Berlin and Bannerjea, 5. August 1932; Letter by University treasury to Liesenberg, 23. August 1932; Letter by Court Cashier’s Office to University, 03. September 1935.

17 Uniarchiv, NS-Dozentenschaft der Friedrich-Wilhelms-Universität Berlin, Dr. Devendra Nath Bannerjea: Letter by Lierau to Engel, 29. July 1935; Letter by Bannerjea to Vice Chancellor, 03. December 1935; Uniarchiv, Personal-Akten des Lehrbeauftragten am Staatswissenschaftlich-Stat. Seminar, Dr. Devendra Nath Bannerjea: Letter by Engel, 11. May 1936; Letter by Bannerjea to Gleispach, 10. January 1937; Letter by University trustee to Bannerjea, 1. April 1937; Letter by Scharschmidt to Reich Minister for Education, 05. June 1939; Letter Reich Minister for Science, Education and National Culture, 20. June 1939; Letter by Harmjanz to University trustee, 01. April 1940.

18 BArch, DS800, A0003: Letter by Schaeder to Reich Ministry of Science, Education and National Culture, 22. January 1935.

19 Uniarchiv, NS-Dozentenschaft der Friedrich-Wilhelms-Universität Berlin, Dr. Devendra Nath Bannerjea: Report on Devendra Nath Bannerjea by Fromme, no date; Letter by Lierau to Steinbeck, 17. May 1935; Letter by Bannerjea to Niedermayer, 12. July 1935; Letter by Lierau to Engel, 29. July 1935; Letter by Bannerjea to Niedermayer, 02. August 1935; BArch, DS800, A0003: Letter by Schultze to Mattiat, 08. October 1934; Letter by Lierau to Mattiat, 08. April 1935.

20 Uniarchiv, NS-Dozentenschaft der Friedrich-Wilhelms-Universität Berlin, Dr. Devendra Nath Bannerjea: Letter by Lierau to Steinbeck, 17. May 1935. “Von Anfang an zeigte er in Italien damals für den Faschismus und dann in Deutschland für die damalige Hitler-Bewegung großes Verständnis. Gerade diese Bewegung interessierte ihn besonders in Hinblick auf ihre philosophischen Grundlagen und die Agrarverhältnisse in Indien.”

21 Uniarchiv, NS-Dozentenschaft der Friedrich-Wilhelms-Universität Berlin, Dr. Devendra Nath Bannerjea: Letter by Steinbeck to Engel, 22. July 1935; Letter by Bieberbach to Bannerjea, 1. August 1935.

22 Uniarchiv, NS-Dozentenschaft der Friedrich-Wilhelms-Universität Berlin, Dr. Devendra Nath Bannerjea: Letter by Bannerjea to Steinbeck, 03. February 1936.

23 BArch, DS800, A0003: Letter by Six to Reich Ministry of Science, Education and National Culture, 11. March 1940.

24 Uniarchiv, Personal-Akten des Lehrbeauftragten am Staatswissenschaftlich-Stat. Seminar, Dr. Devendra Nath Bannerjea: Letter by Frey to University trustee, 09. April 1940; Letter by Frey to University trustee, 21. June 1940; BArchiv DS800, A0003: Letter by Six to Reich Ministry of Science, Education and National Culture, 28. December 1942.

25 Uniarchiv, NS-Dozentenschaft der Friedrich-Wilhelms-Universität Berlin, Dr. Devendra Nath Bannerjea: Letter by Bannerjea to Vice Chancellor, 24. March 1942; Letter by Bannerjea to Six, 14. April 1942; BArch, DS800, A0003: Letter by Schaefer-Rümelin to the Reich Ministry of Science, Education and National Culture, 07. August 1942; Letter by Bannerjea to von Stechow, 08. November 1943; Politisches Archiv des Auswärtigen Amtes (Political Archive of the Foreign Office, hereafter, PA AA), RZ 501/60672: Letter by Bannerjea to Secretary of State Keppler, 18. May 1942.

26 BArch, DS800, A0003: Letter by the Faculty for the Study of Foreign Countries to the Reich Ministry of Science, Education and National Culture, 30. September 1942. See also, BArch, DS800, A0003: Letter to Frey, 24. November 1942.

27 BArch, DS800, A0003: Letter by the Faculty for the Study of Foreign Countries to the Reich Ministry of Science, Education and National Culture, 30. September 1942.

28 BArch, DS800, A0003: Letter by Six to Reich Ministry of Science, Education and National Culture, 20. April 1942.

29 Uniarchiv, Lehrbeauftragter Dr. Bannerjea, Bd. II. B 45: Letter by Groh to Philosophical Faculty University of Berlin, 19. January 1943; Letter by Breloer to Dean, 27. February 1943; Letter Dean to Reich Ministry of Science, Education and National Culture, 01. March 1943; Uniarchiv, Personal-Akten des Lehrbeauftragten am Staatswissenschaftlich-Stat. Seminar, Dr. Devendra Nath Bannerjea: Letter by Frey to University trustee, 19. March 1943.

30 BArch, R/4901/1306; BArch, R/4901/1307. From 1933 until the winter semester 1939–1940, Bannerjea taught on subjects such as ‘Trade unions and cooperatives in India’, ‘The agrarian and economic politics of India’, ‘India’s state structure’, ‘India’s industrial development and the problems of the Indian working class’ and ‘The Indian constitution and the British parliament’. Uniarchiv, Vorlesungsverzeichnisse, Erstes Trimester 1940–Wintersemester 1944/1945. Course titles included ‘The Indian education system’, ‘India: state, volk, culture and economy’, India: religion, philosophy and culture’, India: constitution, administration and society’, ‘India’s industrial development’, ‘The basics of the Indo-Aryan social system’, ‘The intellectual history of the Indian village’ etc.

31 BArch, R/4901/1306: Verzeichnis der Vorlesungen und Übungen im Winter-Semester 1937–1938, Ausland-Hochschule an der Universität Berlin; BArch, R/4901/1307: Verzeichnis der Vorlesungen und Übungen im Winter-Semester 1938–1939, Ausland-Hochschule an der Universität Berlin; Uniarchiv, Vorlesungsverzeichnisse, Erstes Trimester 1941, Sommersemester 1941, Wintersemester 1941/42 and Sommersemester 1942.

32 Uniarchiv, Vorlesungsverzeichnisse, Wintersemester 1941/1942, Sommersemester 1942 and Wintersemester 1942/43.

33 The *Deutsche Akademie*, established in 1925, was a cultural institution aiming to disseminate German language and culture worldwide. In 1928, the *Deutsche Akademie* inaugurated the *Indischer Ausschuß* (India Institute) to support academic exchange between India and Germany by providing scholarships to students, helping to find internships in Germany, promoting studies on India in German universities by inviting Indian scholars and the like. Both the India Institute and the *Deutsche Akademie* increasingly identified with the ideology and policies of National Socialist Germany (see Framke 2013: 66–79; Roy 2021).

34 Without naming it explicitly, Bannerjea was referring to the scholarship programme for Indian students run under the auspices of the India Institute of the *Deutsche Akademie*.

35 BArch, DS800, A0003: Letter by Six to Reich Ministry of Science, Education and National Culture, 20. April 1942.

36 Uniarchiv, Rektorat der Universität Berlin, Habilitationen B, Juli 1937–Dez. 1944, pp. 51–54; Uniarchiv, NS-Dozentenschaft der Friedrich-Wilhelms-Universität Berlin, Dr. Devendra Nath Bannerjea: Letter by Bannerjea to Vice Chancellor, 24. March 1942. No correspondence regarding the submission of Bannerjea’s habilitation nor the manuscript itself is findable in the University Archive Munich (see email correspondence with Dr. Claudius Stein from the Archive with the author, 12. April 2021). Likewise, the manuscript has also not been found in the files of the BArch, the PA AA or the Uniarchiv.

37 PA AA, RZ 229/27504: Copy of the contract between Vowinckel, Bannerjea and Furtwängler, no date; Statement by Alsdorf, 22. November 1943; Letter by State Secretary Keppler to Bannerjea, 10. April 1943; Letter by Bannerjea to Vowinckel, 23. September 1943. Bannerjea’s claim that the Foreign Office functioned as commissioning party seems to have not been totally baseless, as the internal correspondence of members of the Foreign Office indicates their (indirect) involvement in the matter. Besides, the *Sonderreferat Indien* also edited a book series entitled “Indien in Einzeldarstellungen”. Published by Vowinckel, this eight-volume series aimed to provide all necessary information on the subcontinent for those German state agencies dealing with India. Although the contract for the Gandhi biography did not mention the series, it seems likely that the book would have been published as a part of this endeavour (Kuhlmann 2003: 162–163; PA AA, RZ 229/27504: Statement by Kruse, 07. April 1943).

38 PA AA, RZ 229/27504: Statement by Furtwängler 25. January 1943; Statement by Alsdorf, 25. January 1943; Letter by Furtwängler to Bannerjea, 10. April 1943; Letter by Bannerjea to Vowinckel, 28. April 1943; Letter by Kruse to Vowinckel, 24. May 1943; Letter by Vowinckel to Bannerjea, 02. June 1943; Letter by Bannerjea to Vowinckel, 29. July 1943; Letter by Vowinckel to Bannerjea, 07. September 1943; Letter by Bannerjea to Vowinckel, 23. September 1943; Letter by Sarre to Furtwängler, 14. October 1943; Letter by Sarre to Furtwängler, 28. October 1943; Statement by Alsdorf, 22. November 1943; Letter by Sarre to Keppler, 9. May 1944; Letter by Vowinckel to Bannerjea, 31. May 1944; Letter by Alsdorf to Sarre, 19. June 1944; Uniarchiv, Lehrbeauftragter Dr. Bannerjea, Bd. II. B 45: Letter by Bannerjea to Vice Chancellor, 18.10.1944. The publishing house returned the manuscript to Bannerjea in April 1943, but he claimed that three chapters were missing. As Bannerjea had lost the carbon copies of the full manuscript when his flat was bombed a month earlier, he was left with only parts of the manuscript, which were themselves seemingly destroyed during further bombings.

39 Uniarchiv, NS-Dozentenschaft der Friedrich-Wilhelms-Universität Berlin, Dr. Devendra Nath Bannerjea: Letter by Bannerjea to Reich Minister for Science, Education and National Culture, 07. November 1943

40 “Wir sehen also, daß das Verständnis und wechselseitige Interesse rassische und geschichtliche Ursachen hat: Sie beruhen auf der Tatsache, daß beide Länder in der Vergangenheit dem allgemeinen Kulturgute wertvolle Beiträge gegeben haben, und daß sie in Zukunft bestimmt sein können, dem Fortschritt der Menschheit zu dienen. Und wie deutsche Gelehrte bisher zu den Wurzeln altindischen Lebens vorgedrungen sind, und sich in seine Literatur vertieft haben, so wollen wir hoffen, daß die folgende Generation in Deutschland sich mit derselben Liebe zur Sache und mit derselben Gründlichkeit dem Leben und den Problemen des neuen Indien widmen werde.”

41 PA AA, Sig. RZ 207/80562: Letter by Bannerjea to Grobba, 10. September 1931; Letter by Grobba to Bannerjea, 15. September 1931. I am indebted to Ole Birk Laursen who kindly shared this file with me.

42 GandhiServe Archives. URL: www.gandhiserve.net/gandhiserve-archives/ (04. March 2023): Letters by Bannerjea to M.K. Gandhi, 22. September and 15. October 1931; Cable by Desai to Bannerjea, 09. November 1931; Universitäts- und Landesbibliothek, Digitale Sammlungen Darmstadt. Keyserling, Hermann Graf. Nachlass: Korrespondenz A1–Z1, Kasten 087. URL: http://tudigit.ulb.tu-darmstadt.de/show/Keys-087/0039 (06. April 2021): Letter by Hermann Graf Keyserling to Bannerjea, 14.10.1931. For Keyserling’s engagement with India see, Cho (2014); for Furtwängler see, Buschak (2011); and Ahuja (2020).

43 BL, APAC, L/PJ/12/214: Report on Subhas Bose and his associates, 20. September 1933; PA AA, RZ 207/77417: Letter by the Indians’ Student Association to Foreign Office, 30. March 1934. I am indebted to Ole Birk Laursen who kindly shared the second file with me.

44 Sen’s expulsion was rescinded after the Foreign Office intervened. The office feared for Germany’s political and economic interests abroad (Brückenhaus 2017: 176). Sen, who had already left for Britain, was allowed to return to Germany (PA AA, RZ 207/77416: Letter by Sen to Schmidt-Rolke, 17. July 1933). I am indebted to Daniel Brückenhaus who kindly shared this file with me.

45 PA AA, RZ 207/77416: Letter by Bannerjea to Schmidt-Rolke, 11. April 1933; Statement by Mrs Gupta to Hilda M. Bannerjea, on 09. April 1933; Copy of Report, 09. June 1933.

46 Uniarchiv, NS-Dozentenschaft der Friedrich-Wilhelms-Universität Berlin, Dr. Devendra Nath Bannerjea: Letter by Bannereja to Steinbeck, 01. June 1935.

47 Uniarchiv, NS-Dozentenschaft der Friedrich-Wilhelms-Universität Berlin, Dr. Devendra Nath Bannerjea: Letter by Gestapo to University of Berlin, 31. May 1935.

48 PA AA, RZ 229/27504: Letter by Rühle to Secretary of State Keppler, 09. February 1943. For propaganda work of the Foreign Office and the Seehaus unit see, Longerich (1987).

49 PA AA, RZ 229/27504: Letter by Bannerjea to Secretary of State Keppler, 29. May 1942.

50 PA AA, RZ 501/60672: Letter by Bannerjea to Secretary of State Keppler, 18. May 1942. Ein Inder, “der auf Grund seiner Herkunft und Nationalität direkte Kenntnis seines Mutterlandes vermitteln könne, der aber zu gleicher Zeit über die nötige Ausbildung und Kenntnis der wissenschaftlichen Methoden verfüge, die an den deutschen Universitäten üblich sind.”

51 PA AA, RZ 229/27504: Letter to Bannerjea, 09. June 1942; PA AA, RZ 501/60672: Ludwig Alsdorf, Report on the India Institute, 29. May 1942.

52 Archiv der Brandenburgischen Akademie der Wissenschaften (Archive of the Brandenburg Academy of Science), Nachlass Heinrich Lüders, Nr. 120 Deutsch-Indischer Gelehrtenaustausch: Letter by Baron von Putlitz to Reich Minister of Public Enlightenment and Propaganda, 08. July 1933.
